# Climatic adaptation and ecological descriptors of wild beans from Mexico

**DOI:** 10.1002/ece3.4106

**Published:** 2018-06-04

**Authors:** Ivon M. Cerda‐Hurtado, Netzahualcoyotl Mayek‐Pérez, Sanjuana Hernández‐Delgado, José S. Muruaga‐Martínez, Martín A. Reyes‐Lara, Manuel Humberto Reyes‐Valdés, Juan M. González‐Prieto

**Affiliations:** ^1^ Instituto Politécnico Nacional Centro de Biotecnología Genómica Reynosa Mexico; ^2^ Universidad Mexico Americana del Norte Reynosa Mexico; ^3^ Campo Experimental Valle de Mexico INIFAP Coatlinchán Mexico; ^4^ Instituto Tecnológico de Ciudad Victoria Ciudad Victoria Mexico; ^5^ Departamento de Fitomejoramiento UAAAN Saltillo Mexico

**Keywords:** climatic niches, conservation programs, geographical information systems, *Phaseolus* spp., plant genetic resources

## Abstract

Despite its economic, social, biological, and cultural importance, wild forms of the genus *Phaseolus* are not well represented in germplasm banks, and they are at great risk due to changes in land use as well as climate change. To improve our understanding of the potential geographical distribution of wild beans (*Phaseolus* spp.) from Mexico and support in situ and ex situ conservation programs, we determined the climatic adaptation ranges of 29 species and two subspecies of *Phaseolus* collected throughout Mexico. Based on five biotic and 117 abiotic variables obtained from different databases—WorldClim, Global‐Aridity, and Global‐PET—we performed principal component and cluster analyses. Germplasm was distributed among 12 climatic types from a possible 28. The general climatic ranges were as follows: 8–3,083 m above sea level; 12.07–26.96°C annual mean temperature; 10.33–202.68 mm annual precipitation; 9.33–16.56 W/m^2^ of net radiation; 11.68–14.23 hr photoperiod; 0.06–1.57 aridity index; and 10–1,728 mm/month of annual potential evapotranspiration. Most descriptive variables (25) clustered species into two groups: One included germplasm from semihot climates, and the other included germplasm from temperate climates. Species clustering showed 45% to 54% coincidence with species previously grouped using molecular data. The species *P. filiformis*,* P. purpusii*, and *P. maculatus* were found at low‐humidity locations; these species could be used to improve our understanding of the extreme aridity adaptation mechanisms used by wild beans to avoid or tolerate climate change as well as to introgress favorable alleles into new cultivars adapted to hot, dry environments.

## 1 INTRODUCTION

Mexico is a center of origin, diversity, and domestication for many crops of global importance, including beans (*Phaseolus* spp.). The *Phaseolus* genus includes 70–80 species distributed in the Americas, mainly in the Mesoamerican region (central and southern Mexico and Central America) (Gepts, 2014). The genus *Phaseolus* has five domesticated species: *P. vulgaris* L. (common bean), *P. coccineus* L. (“ayocote” bean), *P. lunatus* L. (“lima” bean), *P. acutifolius* Gray (“tepary” bean), and *P. polyanthus* Greenm. (= *P. polyanthus* McFad.) (“acalete” bean). These species include a wide range of cultivated varieties, landraces as well as wild species, some of them endemic (Acosta‐Díaz, Hernández‐Torres, Amador‐Ramírez, Padilla‐Ramírez, & Zavala‐García, 2014; Freytag & Debouck, 2002; Hernández‐Delgado et al., 2015; Hernández‐López, Vargas‐Vázquez, Muruaga‐Martínez, Hernández‐Delgado, & Mayek‐Pérez, 2013).

Climate change is of concern to the scientific community due to the negative impacts on crop production worldwide (González‐Eguiarte et al., 2011; Medina‐García et al., 2016). All predictive scenarios of climate change include region‐specific changes in precipitation as well as increases in temperature and pest and disease incidence (Porch et al., 2013). Wild relatives of crops represent a primary resource for genetic improvement to ensure food security in the face of accelerated population growth and climate change (Debouck, 2000; Gepts, 2014; Porch et al., 2013).

Wild species and local germplasm (e.g., landraces) have favorable alleles that are useful for bean breeding but are generally underused (Acosta‐Díaz et al., 2015; Acosta‐Gallegos, Kelly, & Gepts, 2007); Delgado‐Salinas & Gama‐López, 2015). Genetic variation patterns frequently coincide with environmental variation patterns (Cortés, Chavarro, Madriñán, This, & Blair, 2012). Current research is challenged with collecting, characterizing, and preserving the genetic diversity of wild *Phaseolus* species as well as searching for better strategies for using genetic resources in breeding (Andueza‐Noh, Camacho‐Pérez, Martínez‐Castillo, & May‐Pat, 2016; Bellon et al., 2009; Porch et al., 2013; Ramírez‐Villegas, Khoury, Jarvis, Debouck, & Guarino, 2010; Spataro et al., 2011).

The genetic variability of domesticated *Phaseolus* spp. is well represented in germplasm banks; however, a lack of seeds due to livestock, agriculture, forestry, climate change, or urbanization, among other factors is common (Acosta‐Díaz et al., 2014; Maxted & Kell, 2009; Ramírez‐Villegas et al., 2010). Bean breeders need access to new genotypes for use in the generation of cultivars to satisfy food demand under variable climate or production conditions. Wild parents and domesticated germplasm represent a valuable but underutilized resource (Brozynska, Furtado, & Henry, 2016; Machida‐Hirano et al., 2014).

Eco‐geographical analysis can differentiate collection sites and determine the ranges of adaptation for a species, facilitating collection strategies and supporting in situ conservation (Castañeda‐Álvarez et al., 2016; Khoury et al., 2015; López‐Soto, Ruiz‐Corral, Sánchez‐González, & Lépiz‐Ildefonso, 2005). Agroecological characterization of wild/cultivated niches helps to determine the environmental requirements of a species, providing information on the adaptability and degree of tolerance to specific ecological conditions (Maxted, Dulloo, & Ford‐Lloyd, 2016; Pliscoff & Fuentes‐Castillo, 2011; Svenning, Fløjgaard, Marske, Nógues‐Bravo, & Normand, 2011; Wang et al., 2009). Ecological descriptors are environmental data from collection sites obtained after the standardization of map and layer construction using geographical information systems (GIS) (Cuervo‐Robayo et al., 2014; Suárez‐Venero, Soto‐Carreño, Garea‐Llanos, & Solano‐Ojeda, 2015; Wang et al., 2015). One classification system based on GIS aids in the development of a conservation strategy by enabling the retro‐classification of germplasm collections, facilitating efforts to focus on further exploration, and research in those regions with high probabilities of the presence of specific species or genotypes. This classification system also facilitates the selection of areas for conservation and restoration as well as the prediction of responses to climate change (Elith & Franklin, 2013; Porfirio et al., 2014; Ramírez‐Villegas et al., 2014; Wang et al., 2015).

López‐Soto et al. (2005) characterized climatic distribution types of 25 *Phaseolus* species throughout Mexico and defined environmental intervals where each species grew by itself. However, climate change and population growth accelerate the natural habitat losses and affect species and/or ecosystems diversity (Delgado‐Salinas & Gama‐López, 2015; Maxted, Hawkes, Ford‐Lloyd, & Williams, 1997).

The goals of the present work were (1) to determine the climatic adaptation of 29 *Phaseolus* species from Mexico (particularly those species that represent poorly studied genetic reservoirs), (2) describe their potential geographical distribution, and (3) evaluate differences among the species based on climatic/ecological adaptation descriptors and their comparisons with previously reported genetic descriptors.

## 2 MATERIALS AND METHODS

### 2.1 Germplasm collection

Germplasm collection was conducted from 2012 to 2015 throughout Mexico by José Socorro Muruaga‐Martínez. The taxa were identified in the field according to the descriptors of Piper (1926), Maréchal, Mascherpa, and Stainier (1978), Delgado‐Salinas (1985), Freytag and Debouck (2002) and Salcedo, Arroyave, Toro Ch, and Debouck (2006). Of the 102 accessions obtained, the 29 species and two subspecies included the following: *P*. *acutifolius*,* P. albescens*,* P*. *albiviolaceus*,* P*. *coccineus*,* P*. *esperanzae*,* P*. *filiformis*,* P*. *glabellus*,* P*. *gladiolatus*,* P*. *laxiflorus*,* P*. *leptostachyus*,* P*. *lunatus*,* P*. *maculatifolius*,* P*. *maculatus*,* P*. *macvaughii*,* P*. *micranthus*,* P*. *microcarpus*,* P*. *nodosus*,* P*. *novoleonensis*,* P*. *oligospermus*,* P*. *palmeri*,* P*. *parvifolius*,* P*. *pedicellatus*,* P*. *pluriflorus*,* P*. *purpusii*,* P*. *rotundatus*,* P*. *vulgaris*,* P*. *xanthotrichus*,* P*. *xolocotzii*, and *P*. *zimapanensis* as well as subspecies *P*. *coccineus* subsp. *coccineus,* and *P*. *coccineus* subsp. *striatus* (Appendix S1).

### 2.2 Database construction

The database matrix included information about the collection site of each accession throughout Mexico, including genus, species, subspecies, variety, state, county, latitude, longitude, and elevation. The geographic coordinates of each collection site were projected using datum WGS 1984, after which we obtained the value of each variable from 1950 to 2000. Subsequently, we calculated the climatic ranges for each species as well as the ecological descriptors based on climatic range. Environmental information was obtained using DIVA‐GIS software, version 7.1.7 (Hijmans et al., 2004; http://www.diva-gis.org) based on data presented in Table 1.

**Table 1:** Databases of environmental information used in this study

Spatial variable	Source	Type	Spatial resolution (arc s)	Reference
Minimum, Mean, and Maximum TemperaturePrecipitationElevationBioclimatic variables	WorldClim version 1.4 database	Raster	30	Hijmans, Cameron, Parra, Jones, and Jarvis (2005)
Climate type		Vector	30	Medina‐García et al. (1998)
EvapotranspirationAridity indexRadiation	CGIAR‐CSI database	Raster	30	Zomer et al. (2007), Zomer, Trabucco, Bossio, and Verchot (2008)
Photoperiod	http://www.esrl.noaa.gov/gmd/grad/solcalc/index.html			NOAA (2015)
EcoregionBiome	http://www.conabio.gob.mx/informacion/gis	Vector	62	INEGI, CONABIO & INE (2008)
Soil type				INIFAP & CONABIO (1995)
Vegetation type		Raster	62	CONABIO (2014)
Potential Vegetation		Vector	62	Rzedowski (1990)
Soil moisture regimes		Vector	62	Maples‐Vermeersch (1992)

The climate type base map was calculated using ArcGIS^®^ software by Esri (2011) based on the climatic classification scheme of Instituto Nacional de Investigaciones Forestales Agrícolas y Pecuarias (Medina‐García, Ruiz‐Corral, & Martínez‐Parra, 1998), which included 28 climatic variants for Mexico (Table 2).

**Table 2:** Climatic types of Mexico based on INIFAP‐Mexico classification (Medina‐García et al., 1998)^a^

No.	Climate type	Mean temp. of coldest month (°C)	Number of wet months	Mean annual temp.^b^ (°C)
5	Temperate arid temperate	<5	0 (<30 days)	<5
6	Temperate semiarid temperate	<5	1–3 (30–119 days)	<5
7	Temperate subhumid temperate	<5	4–6	<5
8	Temperate humid temperate	<5	>6	<5
9	Subtropical arid temperate	5–18	0 (<30 days)	5–18
10	Subtropical semiarid temperate	5–18	1–3 (30–119 days)	5–18
11	Subtropical subhumid temperate	5–18	4–6	5–18
12	Subtropical humid temperate	5–18	>6	5–18
13	Subtropical arid semihot	5–18	0 (<30 days)	18–22
14	Subtropical semiarid semihot	5–18	1–3 (30–119 days)	18–22
15	Subtropical subhumid semihot	5–18	4–6	18–22
16	Subtropical humid semihot	5–18	>6	18–22
17	Subtropical arid hot	5–18	0 (<30 days)	22–26
18	Subtropical semiarid hot	5–18	1–3 (30–119 days)	22–26
19	Subtropical subhumid hot	5–18	4–6	22–26
20	Subtropical humid hot	5–18	>6	22–26
21	Tropical arid semihot	>18	0 (<30 days)	18–22
22	Tropical semiarid semihot	>18	1–3 (30–119 days)	18–22
23	Tropical subhumid semihot	>18	4–6	18–22
24	Tropical humid semihot	>18	>6	18–22
25	Tropical arid hot	>18	0 (<30 days)	22–26
26	Tropical semiarid hot	>18	1–3 (30–119 days)	22–26
27	Tropical subhumid hot	>18	4–6	22–26
28	Tropical humid hot	>18	>6	22–26
29	Tropical arid very hot	>18	0 (<30 days)	>26
30	Tropical semiarid very hot	>18	1–3 (30–119 days)	>26
31	Tropical subhumid very hot	>18	4–6	>26
32	Tropical humid very hot	>18	>6	>26

a Mean monthly based on series from 1950 to 2000 (Hijmans et al., 2005).

b Mean annual temperature calculated as the average of mean annual maximum temperature.

### 2.3 Data analysis

Differences across all environmental variables among species were calculated and compared using STATISTICA version 8.0 (StatSoft, 2007) using species as the class variable. Relationships among species were determined by calculating similarity indices using ecological descriptor data (Appendix S2) based on the maximum and minimum values of each parameter. Environmental variables were subjected to principal component analysis (PCA), after which cluster analysis was performed using the complete linkage method and Euclidean distances to identify taxon relationships. The fit of the dendrogram was measured using the *k*‐means clustering algorithm with maximized initial cluster distances.

## 3 RESULTS

**Figure 1:**
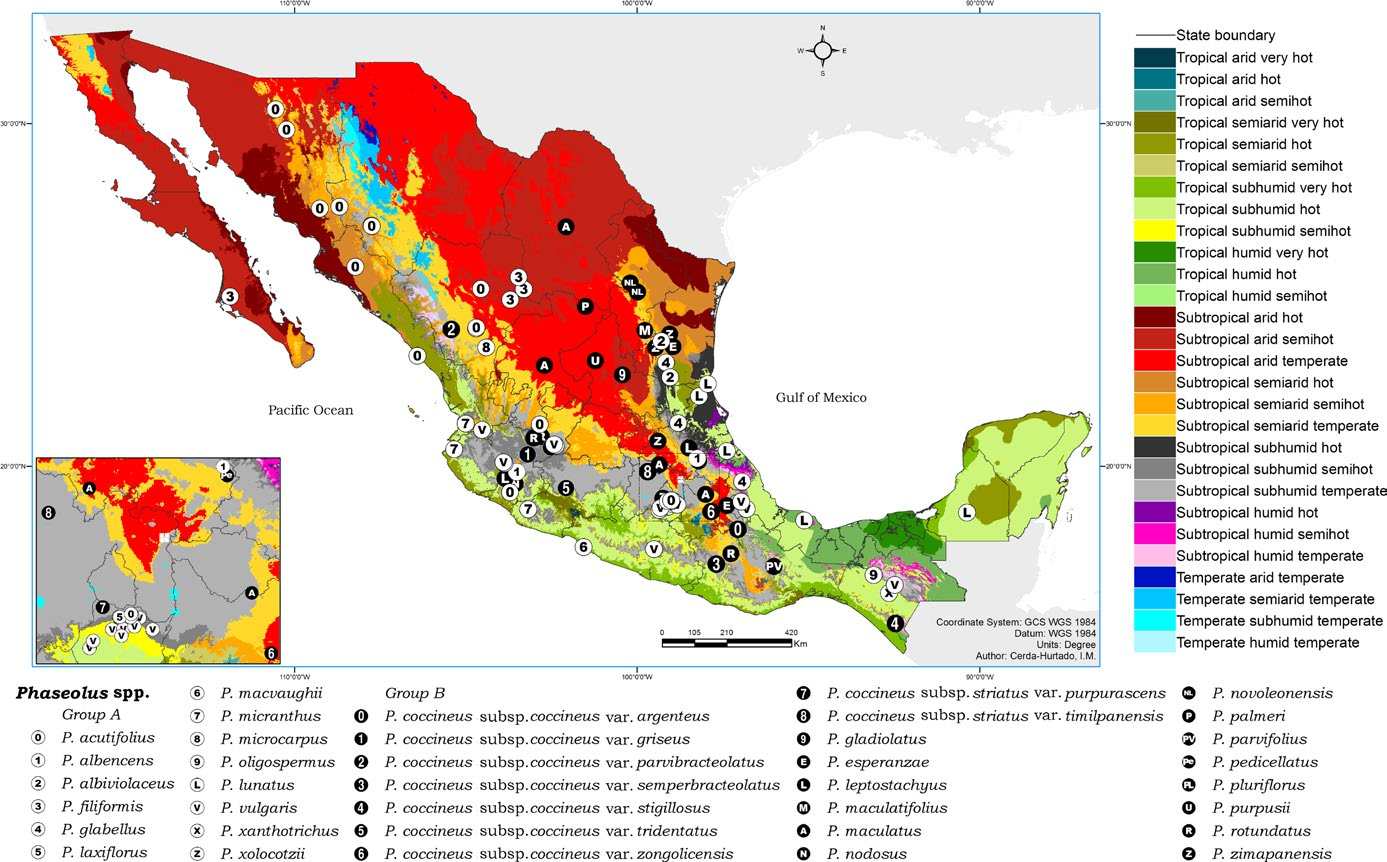
Geographical and climatic distribution of *Phaseolus* spp. in Mexico. State boundaries follow the political divisions of the Estados Unidos Mexicanos. Colors represent the climate types of Mexico calculated following the methodology of Medina‐García et al. (1998). Dots with white backgrounds correspond to Group A, indicating species in the category “subtropical subhumid semihot.” Dots with black backgrounds correspond to Group B, indicating species in the category “subtropical subhumid temperate.” Basemap author: Cerda‐Hurtado, I. M

The geographical distribution of collection sites of the 29 *Phaseolus* species is shown in Figure 1, which includes a broad dispersion of germplasm across regions and climate types of Mexico (12 of 28 climatic variables, Appendix S2).

The collection sites demonstrate the wide eco‐geographical range in elevation and climate variables observed among and within species of the genus, indicating that *Phaseolus* spp. has developed climatic adaptability.

### 3.1 Climatic correspondence

The *Phaseolus* species are more frequently found in subtropical and tropical climates ranging from arid to humid conditions. The subtropical temperate subhumid climate includes the highest number of species and accessions (11 and 20, respectively), followed by subtropical arid temperate (9 and 11, respectively), subtropical subhumid semihot (8 and 20, respectively), and tropical hot‐subhumid (5 and 11, respectively) climates. Accessions of *P. acutifolius* exhibited the highest frequency of accessions and were observed in the following seven climatic types: subtropical semiarid hot, semihot, and temperate; subtropical subhumid temperate and semihot; subtropical arid semihot; and tropical semiarid hot. In terms of the frequency of accessions in different climate types, the species were ordered as follows: *P. coccineus* (subtropical arid temperate; subtropical semiarid semihot and temperate; and subtropical subhumid temperate and semihot), *P. vulgaris* (subtropical subhumid hot, semihot, and temperate; tropical subhumid semihot and hot), and *P. lunatus* (tropical subhumid hot; tropical humid hot and tropical arid very hot).

*Phaseolus* germplasm was found at elevations ranging from eight (*P. lunatus*) to 3,083 m (*P. maculatus*), with a mean of 1,454 m. Below the mean elevation, *P. albiviolaceus*,* P. lunatus*,* P. maculatifolius*,* P. macvaughii*,* P. micranthus*,* P. microcarpus*,* P. oligospermus*, and *P. xanthotrichus* were found. Above the average elevation, *P. acutifolius*,* P. albescens*,* P. coccineus*,* P. esperanzae*,* P. filiformis*,* P. glabellus*,* P. gladiolatus*,* P. laxiflorus*,* P. leptostachyus*,* P. maculatus*,* P. nodosus*,* P. novoleonensis*,* P. palmeri*,* P. parvifolius*,* P. pedicellatus*,* P. pluriflorus*,* P. purpusii*,* P. rotundatus*,* P. vulgaris*,* P. xolocotzii*, and *P. zimapanensis* were found.

The species were distributed between the mean temperatures of 12.1 and 27.0°C, with an annual mean temperature of 19.5°C. One *P. acutifolius* accession from Quiriero, Sonora (northwestern Mexico), was growing below the mean monthly maximum temperature of 40.0°C, and another from the state of Sinaloa (northwestern Mexico) was collected from an area with a mean annual temperature of 34.0°C.

The species *P. acutifolius*,* P. albescens*,* P. albiviolaceus*,* P. coccineus* subsp. *coccineus*,* P. coccineus* subsp. *striatus*,* P. esperanzae*,* P. filiformis*,* P. gladiolatus*,* P. leptostachyus*,* P. lunatus*,* P. maculatifolius*,* P. maculatus*,* P. microcarpus*,* P. nodosus*,* P. novoleonensis*,* P. palmeri*,* P. purpusii*,* P. rotundatus*, and *P. zimapanensis* were distributed among climate types with the lowest precipitation (≤800 mm from May to October and <188 mm from November to April). We found germplasm of *P. acutifolius*,* P. albescens*,* P. albiviolaceus*,* P. coccineus* subsp. *coccineus*,* P. coccineus* subsp. *striatus*,* P. glabellus*,* P. leptostachyus*,* P. lunatus*,* P. maculatus*,* P. micranthus*,* P. oligospermus*,* P. parvifolius*,* P. pedicellatus*,* P. pluriflorus*,* P. vulgaris*, and *P. xanthotrichus* well distributed in high‐precipitation regions (800–1,848 mm from May to October and more than 188 m from November to April). *P. parvifolius* was distributed in high‐precipitation sites (28–337 mm per month), while *P. filiformis* and *P. purpusii* were located in low‐precipitation regions (minimum of 2.5–60.25 mm and maximum of 2.0–69.0 mm).

The annual mean radiation for all *Phaseolus* species was 13.78 W/m^2^ and ranged from 9.33 to 16.56 W/m^2^, whereas the annual mean photoperiod was 12.92 hr of light per day, with a minimum of 11.12 and maximum of 14.96 hr of light per day for *P. acutifolius* in Aconchi, Sinaloa. In general, the germplasm experienced a range of photoperiods, from 11.68 to 14.23 hr of light per day. The mean annual aridity index was 0.58, with a minimum of 0.06 and maximum of 1.57, indicating that *Phaseolus* germplasm is distributed from arid to humid environments. In addition, the annual potential evapotranspiration varied from 10 to 1,728 mm per month, with a monthly mean of 146 mm. The lowest aridity index was found in Comondú, Baja California Sur (0.0596), which is clearly classified as a hyper arid region (Berg et al., 2016) and includes *P. filiformis*. The highest aridity index corresponded to Córdoba, Veracruz (1.57), for *P. vulgaris* and was the most humid location for *Phaseolus*.

### 3.2 Classification

**Figure 2:**
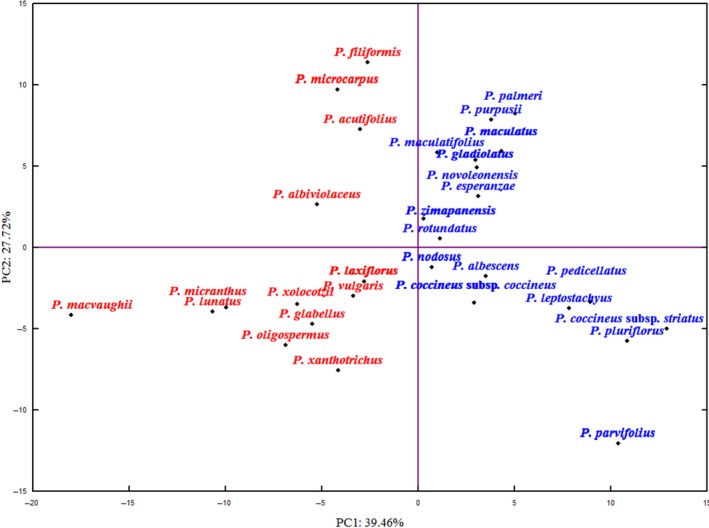
Dispersion of 29 species and two subspecies of *Phaseolus* from Mexico based on the first two principal components of climatic and ecological data. Group A (red) includes species in the category “subtropical subhumid semihot”; Group B (blue) indicates species in the category “subtropical subhumid temperate”

The results of the principal component analysis are presented in Figure 2. The first three PCs explained 78.4% of the total variance (39.5, 27.9, and 11%). PCA identified the 25 most descriptive variables from all 122 measured variables (data not shown); these 25 variables were used to a perform cluster analysis. PC1 showed a correlation of *r* = .99 with the original variable of annual average temperature (Bio1), whereas PC2 had the highest correlation (*r* = .94) with the original variable of average precipitation in June. The complete linkage analysis was consistent with the *k*‐means algorithm and included two cluster categories and given members, such that the means across clusters (for all variables) maximized the differences between them, with a distance between centroids of 1.75 (*k*‐means clustering).

**Figure 3:**
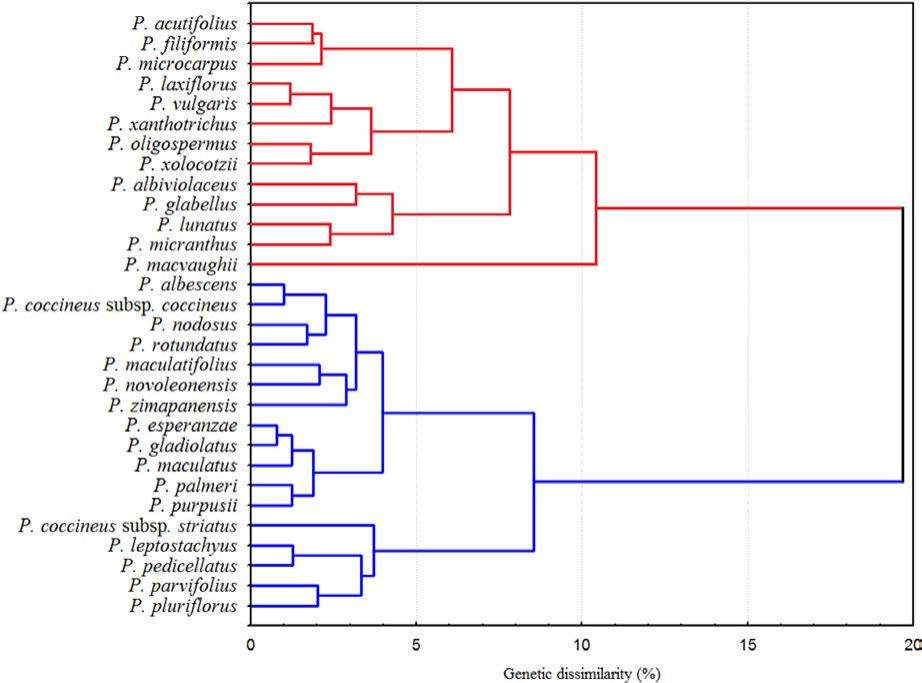
Clustering of 29 species and two subspecies of *Phaseolus* from Mexico, based on Euclidian distances and the single linkage clustering method. Group A (red line) includes species in the category “subtropical subhumid semihot”; Group B (blue line), indicates species in the category “subtropical subhumid temperate”

Both the PCA and cluster analysis (Figure 3) clearly divided the species into two groups. Cluster analysis separated the collection with a linkage distance of 19.68, including Group A, which included species with affinity to hot and semihot environments, and Group B, which included species found in temperate environments.

Group A was named “subtropical subhumid semihot” and included species found at a mean elevation of 1097.7 m and a mean annual temperature of 21.5°C, with an annual precipitation of 992.3 mm (Table 3). In addition, the species of this group are located where the mean precipitation is 35.9 mm during the driest trimester (Bio17). Group A included *P. acutifolius, P. filiformis, P. microcarpus, P. laxiflorus, P. vulgaris, P. oligospermus, P. xolocotzii, P. albiviolaceus, P. glabellus, P. lunatus, P. micranthus*, and *P. macvaughii*. In a previous study based on ribosomal, chloroplastic, and ITS molecular analyses, Delgado‐Salinas, Bibler, and Lavin (2006) assigned to their Group A only five (45%) species, which clustered into group A in the present study. Additionally, *P. glabellus* and *P. microcarpus* were assigned to an unknown group, *P. oligospermus* and *P. xanthotrichus* were assigned to the Tuerckheimii group, and *P. laxiflorus* was assigned to the Pedicellatus group. Delgado‐Salinas et al. (2006) assigned the remaining species: *P. acutifolius* and *P. vulgaris* (group Vulgaris), *P. filiformis* (Filiformis), *P. lunatus* (Lunatus), *P. micranthus* and *P. macvaughii* (Leptostachyus), to their Group B. The species *P. albiviolaceus* and *P. xolocotzii* have not been classified in any previous work.

**Table 3:** Climatic conditions in two groups of *Phaseolus* species from Mexico^a^

Group	Elevation (masl)			Annual temperature (°C)			Rainfall (mm)			Bio17^b^	Bio18^c^
	Min	Max	Mean	Min	Max	Mean	Min	Max	Total		
Group A: Subtropical subhumid semihot	712.1	1460.8	1097.7	14.4	28.6	21.5	9.7	215	992.3	35.9	365.6
Group B: Subtropical subhumid temperate	1855.2	2145.2	1989.3	8.9	23.4	16.1	15.0	181.2	863	51.9	277.8

a Mean monthly based on series from 1950 to 2000 (Hijmans et al., 2005).

b Bio17 = Precipitation of driest quarter.

c Bio18 = Precipitation of warmest quarter.

Group B was named “subtropical subhumid temperate” and included those species growing to a mean elevation of 1989.3 m under environments with a mean annual temperature of 16.1°C and annual precipitation of 863 mm (Table 3). The locations of the species in this group have a mean precipitation of 51.9 mm during the driest trimester (Bio17). This group included *P. albescens*,* P. coccineus* subsp. *coccineus*,* P. nodosus*,* P. rotundatus*,* P. maculatifolius*,* P. novoleonensis*,* P. zimapanensis*,* P. esperanzae*,* P. gladiolatus*,* P. maculatus*,* P. palmeri*,* P. purpusii*,* P. coccineus* subsp. *striatus*,* P. leptostachyus*,* P. pedicellatus*,* P. parvifolius,* and *P. pluriflorus*. Six of these species (54%) were assigned to Group B, as cited by Delgado‐Salinas et al. (2006): *P. albescens*,* P. parvifolius, P. coccineus* subsp. *coccineus*, and *P. coccineus* subsp. *striatus* (group Vulgaris); *P. leptostachyus* (Leptostachyus); and *P. maculatus* (Polystachios). We note that Delgado‐Salinas et al. (2006) assigned to Group A the species *P. pluriflorus*,* P. gladiolatus*, and *P. zimapanensis* (Tuerckheimii) as well as *P. pedicellatus* and *P. esperanzae* (Pedicellatus). The species *P. purpusii*,* P. palmeri*,* P. novoleonensis*,* P. maculatifolius*,* P. rotundatus,* and *P. nodosus* have not been described in previous reports.

## 4 DISCUSSION

We identified the distribution of 29 species and subspecies of *Phaseolus* across diverse climatic and ecological conditions in Mexico. We found this genus in 12 climatic types, whereas López‐Soto et al. (2005) reported that *Phaseolus* was distributed throughout 26 climatic types. We analyzed fewer accessions and species than López‐Soto et al. (2005) and worked only with “fresh” collections obtained under field conditions without the use of herbariums or other databases. However, this work provides new information about uncharacterized species, such as *P. albescens*,* P*. *albiviolaceus*,* P*. *gladiolatus*,* P*. *laxiflorus*,* P*. *macvaughii*,* P*. *nodosus*,* P*. *palmeri*,* P*. *parvifolius*,* P*. *purpusii*,* P*. *rotundatus*, and *P*. *xolocotzii* (Acosta‐Díaz et al., 2015; Delgado‐Salinas & Gama‐López, 2015).

The grouping of *P. microcarpus* with *P. acutifolius* and *P. filiformis* in this work contradicts previous studies based on DNA sequences (Delgado‐Salinas et al., 2006). Our data agree with the environmental characteristics of the collecting sites of these species (Appendix S2).

Successful dispersion across diverse environmental conditions of some species, such as *P. acutifolius*,* P. coccineus*,* P. lunatus*, and *P. vulgaris*, coincide with their domestication patterns throughout Mesoamerica. Conservation of these species is a priority because of their importance in breeding programs (Andueza‐Noh et al., 2016; De Ron et al., 2015; Debouck, 2000; Rodriguez et al., 2015; Shi & Lai, 2015; Singh, Singh, & Dutta, 2014). In addition, further work is needed to reinforce knowledge regarding population genetics and the response to ecological or climate changes and to evaluate survival rates (Delgado‐Salinas & Gama‐López, 2015). Recently, Bitocchi et al. (2017) reported that domestication increases the functional diversity of target loci, which could be used to control traits related to expansion and adaptation to new agroecological growing conditions. Zoro Bi et al. (2003) detected genetic variability primarily at the interpopulation level, with low values for allelic richness, expected heterozygosity, and interpopulational gene flow in wild *P. lunatus* from Central Valley, Costa Rica. These authors recommended protection of *P. lunatus* populations at each distinct ecologic site, regardless of size, to preserve unique alleles and other significant morphological or physiological traits (Barrantes, Macaya, Guarino, Baudoin, & Rocha, 2008).

Some species are present in multiple climatic types, but their populations are small; despite this, these species have persisted for thousands of years. However, they may be at risk due to changing climatic conditions and anthropogenic factors. Thus, species such as *P. gladiolatus*,* P. laxiflorus*,* P. micranthus*,* P. novoleonensis*,* P. pluriflorus*, and *P. zimapanensis* need special attention in terms of study and conservation (Acosta‐Díaz et al., 2015; Delgado‐Salinas & Gama‐López, 2015).

Other species with a restricted distribution (no more than one or two climatic types) could be influenced by sample size, and new information concerning some species was obtained. For example, *P. esperanzae* was located in a subtropical arid semihot climatic type near Ciudad Victoria, Tamaulipas (northeastern Mexico); *P. maculatus* was detected near Tepatlaxco de Hidalgo, Puebla (central highlands), in a subtropical subhumid temperate climate; and *P. xanthotrichus* was found growing in a tropical subhumid semihot climate in Teopisca, Chiapas (southeastern Mexico). These findings are preliminary due to the low number of samples analyzed, although they provide valuable information for future and more intensive collections (Gil & Lobo, 2012; Pliscoff & Fuentes‐Castillo, 2011; Ramírez‐Villegas et al., 2010; Russell et al., 2016).

One major trait for selection and breeding in *P. vulgaris* is the photoperiod insensitivity of new cultivars. The photoperiod insensitivity trait allows *P. vulgaris* plants to be cultivated at various latitudes at Mexico (Peña‐Valdivia, Aguirre‐Rivera, & Arroyo‐Peña, 2012) and allows phenology synchrony beginning with the emergence of seedlings (Peña‐Valdivia, Trejo, Celis‐Velazquez, & López‐Ordáz, 2013). A major trait of wild beans, however, is that the seeds are tolerant to less stringent storage conditions; they are capable of withstanding high temperatures and high relative humidity without germinating (dormancy). Therefore, seeds of wild beans can be stored for months or years under natural environmental conditions and remain dormant (Celis‐Velazquez, Peña‐Valdivia, Luna‐Cavazos, & Aguirre‐Rivera, 2010; Peña‐Valdivia et al., 2013). Dormancy is related to seed coat hardness, although some *P. vulgaris* accessions from the state of Durango (semiarid central plateau) spontaneously germinated under semiarid climate conditions at 1,820 m, where temperatures are extreme and precipitation periods are brief during the summer (López‐Herrera, Aguirre‐Rivera, Trejo, & Peña‐Valdivia, 2001).

We found broad climatic availability of *Phaseolus* based on elevation and climatic variable ranges of the germplasm. The mean elevation (1,453 m) was lower than that reported by López‐Soto et al. (2005) (1,900 m) because some species were collected closer to sea level, although the maximum and minimum ranges were similar. The data suggest that species with a reduced range in elevation or distribution in the highest elevations (*P. coccineus* subsp. *coccineus*,* P. gladiolatus*,* P. palmeri*, and *P. pedicellatus*) will face adaptation obstacles under climate change, with a consequent loss of genetic diversity (Hill, Griffiths, & Thomas, 2011). In addition, the data may predict the presence of proper germplasm for breeding based on elevation adaptation (Porch et al., 2013).

No species were found at locations with maximum temperatures lower than the annual mean of all *Phaseolus* species (19.5°C), although some species were found at sites with minimum temperatures higher than general mean temperatures. For example, *P. lunatus* and *P. macvaughii* were collected from sites with the lowest elevation (8 and 14 m, respectively), but both species have adapted to coastal regions with high temperatures all year (Andueza‐Noh et al., 2016; Martínez‐Castillo, Camacho‐Pérez, Villanueva‐Viramontes, Andueza‐Noh, & Chacón‐Sánchez, 2014; Meza‐Vázquez, Lépiz‐Ildefonso, López‐Alcocer, & Morales‐Rivera, 2015). The most optimistic climate change scenario will involve an increase in temperature. Therefore, we suggest the necessity to study adaptation patterns and ranges to predict the future distribution of species; the reduced range of temperatures will reduce adaptation capacity (Hill et al., 2011; Porch et al., 2013). In addition, as high temperatures are associated with an increase in pest and disease incidence, we can efficiently breed beans for increased resistance to these adverse factors if we know the environmental conditions that act as selection pressure sites (Abberton et al., 2015; Miklas, Kelly, Beebe, & Blair, 2006).

The best‐studied stress in bean breeding is drought stress (Beebe, Rao, Blair, & Acosta‐Gallegos, 2013; Blair, Cortés, & This, 2016; Miklas et al., 2006; Rodriguez et al., 2015; Villordo‐Pineda, González‐Chavira, Giraldo‐Carbajo, Acosta‐Gallegos, & Caballero‐Pérez, 2015). The most widely used strategy consists of the identification of wild and landrace germplasm resistant to drought stress (Cortés, Monserrate, Ramírez‐Villegas, Madriñán, & Blair, 2013; Porch et al., 2013). Some traits in wild *P. vulgaris* accessions have been associated with resistance to water stress. For example, root anatomy changes (low reductions in parenchyma cells and cell division compared with domesticated genotypes) (Peña‐Valdivia et al., 2010) as well as physiological and biochemical changes in root tissue can occur to provide tolerance to dehydration (Sánchez‐Urdaneta et al., 2003).

The species *P. filiformis* is known for its strong adaptation to arid and saline environments (Bayuelo‐Jiménez, Debouck, & Lynch, 2002; Delgado‐Salinas & Gama‐López, 2015; Delgado‐Salinas, Turley, Richman, & Lavin, 1999; López‐Soto et al., 2005; Porch et al., 2013). Other noteworthy accessions in this regard were found, such as *P. maculatus* from Cuatro Ciénegas, Coahuila, where precipitation is 18.5 mm per month, and *P. purpusii* from Charcas, San Luis Potosí, with 30.3 mm of precipitation per month. Both species may be useful for analyzing and understanding the mechanisms of resistance to low water stress (Delgado‐Salinas & Gama‐López, 2015; Delgado‐Salinas et al., 2006; Hernández‐Delgado et al., 2015; Redden et al., 2015; Singh et al., 2014). In this regard, drought resistance genes have been reported in *P. maculatus* (Lioi et al., 2007).

The PCA and cluster analysis provided similar results. In addition, species grouped by climatic and ecological variables were 45% to 54% identical to those of the molecular groups (ITS and *trn*K sequences) described by Delgado‐Salinas et al. (2006). Differences in grouping by the two strategies reinforce the necessity to complement eco‐geographical data with phenotypic and/or genotypic information, as morphologic, biogeographic, or ecologic distinctness can be detected even in phylogenetically related species (Delgado‐Salinas & Gama‐López, 2015).

Our goal is to gain a better understanding of the distribution of wild populations, which can greatly improve decision making with respect to the management and use of genetic resources (Parra‐Quijano, Iriondo, & Torres, 2012; Thormann et al., 2015; Tohme, Beebe, & Iglesias, 1999). Similar results have been reported in *P. lunatus* from Costa Rica (Degreef, Rocha, Vanderborght, & Baudoin, 2002; Vargas, Castro, Macaya, & Rocha, 2003; Zoro Bi, Maquet, & Baudoin, 2003) and Mexico (Andueza‐Noh et al., 2016; Martínez‐Castillo, Zizumbo‐Villarreal, Perales‐Rivera, & Colunga‐GarcíaMarín, 2004) as well as in other species, such as *Zea mays* (Ruiz‐Corral et al., 2008), *Sorghum bicolor* (Iqbal et al., 2010; Nkongolo & Nsapato, 2003), *Theobroma cacao* (Suárez‐Venero et al., 2015), and *Crataegus* spp. (Núñez‐Colín et al., 2008).

Wild germplasm is the primary genetic resource for plant breeding (Castañeda‐Álvarez et al., 2016; Piñero et al., 2009). Modern tools such as genomics can provide information for the use and management of genetic resources to compensate for the limited variation associated with crop domestication. Therefore, breeders can overcome interspecific barriers to exploit gene traits from wild germplasm throughout a particular genus (Abberton et al., 2015; Brozynska et al., 2016; De Ron et al., 2015; Estrada, Guillén, Olivares, Díaz, & Alvarado, 2007; Kole et al., 2015; Rendón‐Anaya et al., 2017).

The use of environmental information in breeding programs has been promoted by the use of methodologies that can predict the presence of specific traits in germplasm growing at specific locations with consequent savings of time and cost (Cortés et al., 2013; Parra‐Quijano et al., 2012; Rodriguez et al., 2015; Song et al., 2015; Thormann et al., 2015; von Wettberg, Marques, & Murren, 2016). However, conservation programs are not considered important by the government and have therefore not received economic support, resulting in the low representation of wild crop relatives in germplasm banks (Castañeda‐Álvarez et al., 2016; Maxted et al., 2016). In addition, it is necessary to know the conditions of in situ conservation to conduct long‐term monitoring because of the advantages of low maintenance costs and the dynamic evolution of populations that this type of conservation offers (Acosta‐Díaz et al., 2015; Ramírez‐Villegas et al., 2014; Smýkal et al., 2015). Furthermore, we must consider the threats to wild germplasm caused by agriculture, urbanization, invasive species, contamination, mining, and climate change. Climate change is of concern to the scientific community due to the negative impacts on crop production worldwide (González‐Eguiarte et al., 2011; Medina‐García et al., 2016) and the short time available for wild species to adapt (Castañeda‐Álvarez et al., 2016; Londoño‐Murcia, Tellez‐Valdés, & Sánchez‐Cordero, 2010; Porfirio et al., 2014; Redden et al., 2015; Yadav et al., 2011).

This work provides information about the diversity of the climates adapted to by wild *Phaseolus* germplasm from Mexico, emphasizing the need to include the poorly studied genetic reservoirs and to use the recently collected accessions. Our results reinforce the knowledge regarding diversity and gene flow among *Phaseolus* species from Mexico with an emphasis on the wild populations with a restricted distribution endangered by climate change and the factors that threaten genetic resource availability (destruction of ecosystems, agriculture, and urbanization) and the loss of populations (Acosta‐Díaz et al., 2014, 2015; Porch et al., 2013). Germplasm distribution is closely associated with forest distribution. Thus, the conservation of forest resources is a major challenge for Mexico in terms of combating climate change and improving the conservation of valuable genetic resources. Most of the conservation and use of forest resources in Mexico are the responsibility of indigenous ethnicities, responsibility that it is inherited from generation to generation (Boege, 2008; Pretty et al., 2009). We suggest that it is imperative to legitimize and strengthen community property to support genetic resource management and conservation (Maffi, 2005; Naughton‐Treves & Wendland, 2014). Finally, we propose future work to model the current potential distribution of *Phaseolus* spp. in Mexico and to evaluate the impact of climate change on their future distribution.

Such information would aid in decision making to implement conservation strategies for vulnerable genetic resources, especially those around the proposed critical area of domestication.

## 5 CONCLUSIONS

Wild crop relatives represent a primary genetic resource in crop improvement to ensure food security in the face of accelerated population growth and climate change. The eco‐geographical analysis of germplasm collection sites in Mexico revealed the broad dispersion and distribution of wild *Phaseolus* based on elevation, mean annual temperature, precipitation, and photoperiod patterns. Our results confirm the broad climatic variability adaptation of *Phaseolus* and represent the potential geographical distribution of these species.

The *Phaseolus* species studied were abundant in climates with arid to humid conditions, especially in subtropical and tropical environments. The highest species diversity was found in subtropical temperate subhumid climate types. *P. acutifolius* was the most frequently observed species and was found in seven climatic types.

Knowledge of the climatic distribution supported by geographical information systems will allow us to generate maps and establish potential areas of distribution, adaptation, and location of wild *Phaseolus* germplasm in Mexico. These data will assist the planning of future collection expeditions and allow efficient strategies to acquire, manage, and support in situ conservation of wild bean genetic resources.

## CONFLICT OF INTEREST

None declared.

## AUTHOR CONTRIBUTION

Ivon M. Cerda‐Hurtado‐Corrresponding author. Netzahualcoyotl Mayek‐Pérez‐Served as scientific advisor. Sanjuana Hernández‐Delgado‐Served as scientific advisor. José S. Muruaga‐Martínez‐Provided study plants. Martín A. Reyes‐Lara‐Provided collected data. Manuel H. Reyes‐Valdés‐Critically reviewed the study proposal. Juan M. González‐Prieto‐Critically reviewed the study proposal.
